# Associations between psycho-behavioral risk factors and diabetic retinopathy: NHANES (2005–2018)

**DOI:** 10.3389/fpubh.2022.966714

**Published:** 2022-09-15

**Authors:** Xiao-Jia Sun, Guo-Heng Zhang, Chang-Mei Guo, Zi-Yi Zhou, Ya-Li Niu, Ling Wang, Guo-Rui Dou

**Affiliations:** ^1^Department of Ophthalmology, Eye Institute of Chinese PLA, Xijing Hospital, Fourth Military Medical University, Xi'an, China; ^2^Department of Health Statistics, Faculty of Preventive Medicine, Fourth Military Medical University, Xi'an, China

**Keywords:** diabetic retinopathy, depression, sleep duration, NHANES, recreational activity

## Abstract

**Introduction:**

Diabetes mellitus (DM) and diabetic retinopathy (DR) increase the global burden. Since their pathogenesis is complex, it is necessary to use the biopsychosocial model to discover the most effective strategies. The study is aimed to investigate the psycho-behavioral factors of DR and confirm the discrepancies from previous studies.

**Research design and methods:**

The study comprised seven cycles of cross-sectional data of the National Health and Nutrition Examination Survey (NHANES) from 2005–2006 to 2017–2018. Samples of DM were selected from this complex multi-stage probability sample and divided into the non-DR and DR groups, where 4,426 samples represented 18,990,825 individuals after weighting. This study comprehensively explored the biological, social, and psychological risk factors of DR, among which the biological factors included blood pressure, blood routine, HbA1c%, blood glucose, the duration of DM, family history, comorbidities, and treatment methods. Social aspects include gender, education, income, insurance, smoking, drinking, sleep habits, and recreational activities. The Patient Health Questionnaire-9 (PHQ-9) was used to assess the psychological state. Taylor series regression was used to examine the connection between factors and DR.

**Results:**

Men accounted for 55.5% of the DR group (*P* = 0.0174). Lymphocyte count, insulin treatment, heart failure, stroke, liver condition, and renal failure showed significant differences in DR (*P* < 0.05). The incidence of depression in DR was 40.5%. Mild to moderate depression [odds ratio was associated with DR [(OR) = 1.37, 95% confidence interval (CI): 1.06–1.79], but there was no statistical difference in severe depression (OR = 1.34, 95% CI: 0.83–2.17). Although ≤ 6 h of sleep was associated with DR (OR = 1.38, 95% CI: 1.01–1.88), we found no statistical differences in alcohol consumption, recreational activities, or sedentary time between the two groups in our current study (*P* > 0.05).

**Conclusions:**

The biological risk factors of DR are significant. It showed that stroke is associated with DR, and retinal exams have the potential value as a screening tool for the brain. Besides, psycho-behavioral risk factors of DR should also be paid attention. Our study highlights that mild and moderate depression and ≤6 h of sleep are distinguishably associated with DM complicated with DR. It indicates that psycho-behavioral risk factors confer a vital influence on diabetic health care and DR.

## Introduction

Diabetic retinopathy (DR) is one of the most prevalent microvascular complications of diabetes mellitus (DM) and a leading cause of blindness globally (1). With the global incidence of DM quadrupling over the past four decades (2), the visual impairment caused by DM has snowballed. According to reports, the global prevalence of DR is 34.6%, with 1 in 10 people suffering from sight-threatening DR (3). Apart from vision loss, DR also signifies a heightened risk of life-threatening systemic vascular complications (4) and causes a significant financial burden (3), making DR a serious public health problem. For instance, the number of DR is predicted to reach 16 million by 2050, and diabetes-related vision loss is expected to cost US $500 million annually (2).

The DR Barometer Report Global Findings 2020 (5) estimated that seven out of ten individuals with diabetic-related ocular complications had experienced days of poor physical and mental health. Increasing studies implied that patients with DR are prone to depression, loss of confidence, and other adverse emotional reactions (6, 7) and behaviors (8). Is there a positive correlation between psycho-behavioral risk factors and DR?

Prior research established that DM is more likely to suffer from depression (9), and the occurrence of DR accompanied by psychopathy, particularly depression, is growing year by year. In Australia, vision-threatening DR and moderate or severe vision impairment were considered independent risk factors for increased depressive symptoms in adults with DM (10). According to several studies, depression is linked to unhealthy behaviors, lack of exercise, and neuroendocrine changes, all of which may accelerate the progression of DM and its complications (11, 12). The progression of chronic diseases is often a process of mutual influence and interaction of biopsychosocial factors. The co-occurrence of a psychiatric condition and unhealthy behaviors are related to worse glycemic control, higher incidences of poor metabolic outcomes, and a higher risk of complications in DM. Tobacco or alcohol consumption, lack of physical activity, sedentary lifestyle (13), poor medication adherence, and self-management all aggravate retinopathy (14). Notably, potential psychological stress could accelerate the progression of DR through common biologic pathways (8). As a result, the American Diabetes Association (ADA) suggests a routine screening for depression in patients with diabetics (15). It also suggests that it is necessary to pay attention to the influence of psycho-behavioral factors on DM and its related visual impairment.

However, due to the relatively small sample size, specialized populations or hospitals, and short follow-up, the research on the exact association between psycho-behavioral factors and DR is still limited, and the results are controversial (16). A systematic review concluded that the incidence and progression of DR had a bidirectional relationship with depression (7), though others disagree (17). Other studies showed that DR had no effect on depression (18–21) and health-related quality of life scores in patients (19, 22). Hence, whether depression is a risk factor for DR and whether behavioral patterns reduce the risk of DR deterioration remain to be fully explored.

Therefore, we updated the incidence of DR and depression among DM in the US population and analyzed the risk factors of DR based on the data published in the NHANES 2005–2018. We aimed to enhance the psycho-behavioral assessment of DR by revealing the association between DR and psychological state and behavioral factors, which would provide further concern in DR screening guidelines to minimize the rate of DR-related blindness and improve the quality of life (3).

## Research design and methods

### Study population

The National Health and Nutrition Examination Survey is a national cross-sectional survey that represents the non-institutionalized civilian resident US population and is distinguished by its complex sampling strategy. Data are collected from a home interview and standardized physical mobile examination centers (MECs) released in 2-year cycles. The National Center approved the study procedures of the Health Statistics Research Ethics Review Board. Participants were given informed consent before any data was collected and the NHANES protocol details are available in the website of the Centers for Disease Control and Prevention (CDC) (23). We applied seven cycles from 2005 to 2018 to assess the association between clinical, psychological, and behavioral factors and diabetic visual impairment. Respondents aged 18 or older with DM were selected. Patients with incomplete depression screening questionnaires and pregnant women during the interview were excluded.

### Assessment of DM and diabetic visual impairment

Diabetes was defined as having a fasting plasma glucose (FPG) level of more than 126 mg/dL or a glycated hemoglobin (HbA1c) level of at least 6.5% or having a physician-diagnosed diagnosis of DM (13, 24, 25). Diabetic visual impairment was confirmed using a dichotomous, self-reported item, indicating that a doctor had informed the respondent that diabetes had affected their eyes.

### Assessment of biological factors

We focused on the diabetic-related clinical variables of DR, encompassing the family history of DM, the duration of DM, the last time of the dilated eye examination, the frequency of self-monitoring blood glucose, the level of blood glucose control, and the therapy of DM. Body mass index (BMI) was computed by dividing kilogram weight by height in meters squared. Urine albumin and creatinine levels were measured with a fluorescent immunoassay and the Jaffe rate reaction method, respectively.

We chose the presence of comorbidities based on previous research: (1) hypertension (2) hypercholesterolemia; (3) heart disease covering congestive heart failure (CHF), coronary heart disease (CHD), angina, and myocardial infarction (MI); (4) stroke; (5) cancer (any); (6) renal failure; and (7) hepatic failure.

### Assessment of psychological factor

The psychological status of patients has been assessed using a scale for 14 years. The Patient Health Questionnaire-9 (PHQ-9), as one of the scales for depression state, can assess the psychological state of patients to a certain extent and is regarded as a unified way and method to measure the psychological state of patients by the database (26). PHQ-9 adds the scores of each item and ranges from 0 to 27. In fact, 5, 10, 15, and 20 points represent thresholds demarcating the lower limits of mild, moderate, moderately severe, and severe depression. A score between 0 and 4 is considered normal, and scores higher than 15 signify a possible clinical level of depression (26). Subsequently, the depression categories were collapsed into three groups: no depression, mild or moderate depression, and major depression in this setting.

### Assessment of social factors

In addition to gender, education, income, and insurance status, the social factors also include indicators of smoking, drinking, sleeping, and exercise habits of patients with DM. Smoking and drinking were classified according to the questionnaire. The NHANES guidance defines recreational activities as those lasting longer than 10 min per week and that do not include exercise caused by work or traffic. Recreational activities are further classified as vigorous recreational activities or moderate recreational activities. High-intensity activities, such as running and basketball, can produce breathing and an increase in heart rate, but moderate-intensity exercises, such as walking, cycling, or swimming, cause only slight breathing and an increase in the heart rate. In addition, high-intensity exercise corresponded to two times the moderate-intensity exercise score, which was surveyed by uniformly trained professional interviewers. We collected data about the duration of different types of recreational activities, sedentary time, and sleeping habits. Moreover, we paid attention to both physician-diagnosed and self-reported sleeping disorders.

### Assessment of covariates

We analyzed the risk factors related to DR as comprehensively as possible from the biological, psychological, and social perspectives in univariate analysis.

Clinical biological indicators, together with traditional social indicators, should be included as confounding background factors in the multivariate analysis from the biopsychosocial model, with a focus on the long-term impact of psychological and social behavior factors on chronic diseases. In other words, clinical biological factors should be used as a baseline and gradually calibrated, allowing for a more accurate comparison of indicators of psychological and social-behavioral factors between the DM and DR groups.

A household interview was undertaken to get information such as age, gender, race, marital status, education, and income. The non-Hispanic Asian subgroup was not available before 2011 due to the survey design; individuals were categorized into five groups (27). Thus, we added the extra group for Non-Hispanic Asians into the fifth group to keep the research consistent.

### Statistical analysis

Our statistical analysis was divided into three parts to investigate the connection between risk factors and DR. First, the participants were divided into two groups based on whether or not they had DR. Differences in baseline characteristics between the groups were compared *via t*-tests in continuous variables and χ2 tests in categorical variables. They were presented as mean ± standard deviation (SD) and frequency or percentage. The complex sample was then analyzed using univariate and multivariate logistic regression models (28). The variance estimation was performed to determine the relationship between factors and DR. Finally, based on the results of the previous step, four multiple regression models with expanding adjustment were used to estimate the odds ratios (ORs) and 95% confidence intervals (CIs) for examining relationships. Additionally, multiple imputations were employed to account for missing data (since triglyceride and cholesterol deficiency rates were >25%, these variables were excluded).

Notably, this complex sampling (23) includes stratified, cluster, multistage sampling, and unequal probability proportional to a measure of size (PPS), and this sampling weight needs to be considered. On the one hand, this design makes it possible to merge more cycles and enables more excellent statistical reliability (WTMEC2YR/7). On the other hand, traditional regression will lead to wrong inference conclusions. In particular, the standard error and CI of parameter estimates may be seriously underestimated, and the probability of class I error in hypothesis testing is much higher. Therefore, the existing research (11, 13, 27) and NHANES tutorials recommend SURVEYMEANS, SURVEYREG, and SURVEYLOGISTIC (29) to achieve statistical description and complex sampling logistic regression analysis. Statistical analysis was executed with SAS 9.4 (SAS Institute, Cary, North Carolina).

## Result

### Characteristics of participants

**Figure 1:**
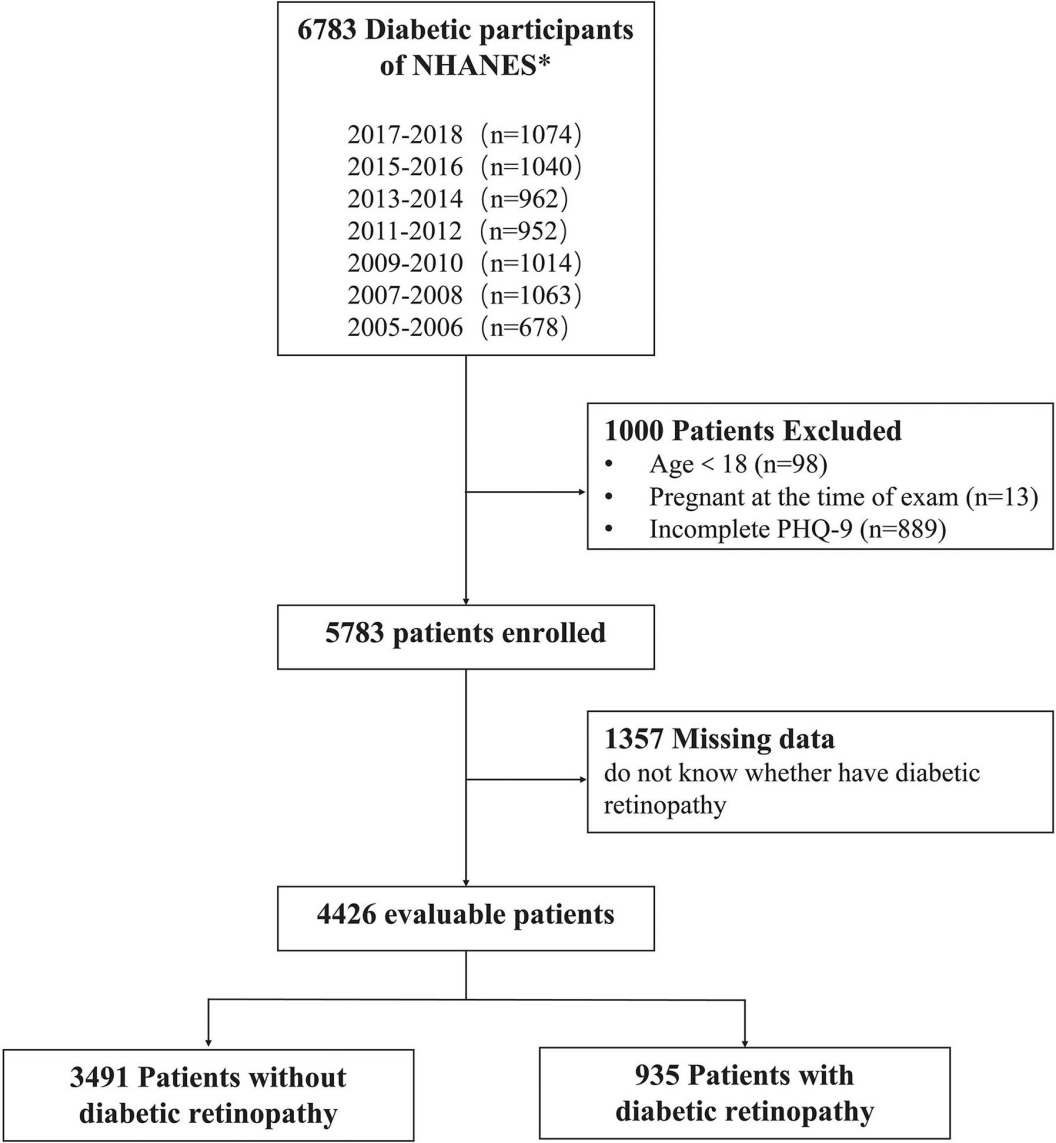
Flow diagram of the study population's inclusion and exclusion criteria. 2005–2018 National Health and Nutrition Examination Survey (NHANES).

About 6,783 patients with DM were enrolled in the NHANES from 2005 to 2018 (Figure 1), excluding those under the age of 18 (*n* = 98), who were pregnant (*n* = 13), and with incomplete PHQ-9 data (*n* = 889). Among the 5,783 initially enrolled respondents, 1,357 were removed due to missing data on whether they had DR. Ultimately, 4,426 unweighted samples were included in the analysis, representing 19 million non-institutionalized US population.

We made the following statistics on the missing values in the final 4,426 samples: missing data were found for education (*n* = 9 [0.1%]), income (*n* = 473 [9.8%]), PIR (*n* = 431 [9.0%]), alcohol consumption (*n* = 14 [0.2%]), family history of DM (*n* = 112 [2.4%]), pupils dilated exam (*n* = 37 [0.7%]), treatment of DM (*n* = 8 [0.1%]), and sleeping trouble (*n* = 18 [0.6%]); none of the missing data of the comorbidities were >1%. Moreover, there were missing values in the duration of DM and frequency of self-monitoring blood glucose (*n* = 41 and 28, respectively). Missing data in the categorized variables are grouped separately, and the totals are unweighted.

### Characteristics of variables

Eligible DM was divided into two groups according to visual impairment, with a total of 935 (21.8%) patients having impaired eyes. Selected characteristics were comparable in both groups, and all were weighted proportions by SURVEYMEANS and SURVEYREG modules in SAS 9.4 (29, 30).

The four parts of the risk factors are summarized in Table 1, (more details can be seen in Supplementary Tables 1–4). The mean ages for DM without and with DR were 60.6 (SE, 0.8) and 61.0 (SE, 1.3) years, respectively. Participants with DR were predominately to be men (55.5% vs. 48.8%, *P* = 0.0174) and had a lower poverty/income ratio (35.7% vs. 29.4%, *P* = 0.0322). Although there was no significant difference in insurance coverage between them, the number of private insurance purchases was statistically significant (*P* = 0.0019). In contrast, we did not find significant differences in race, marital status, education level, and income on the sociodemographic part of the baseline.

**Table 1:** Baseline characteristics of diabetes in NHANES.

**Variables**	**Total**	**Diabetes without DR**	**Diabetes with DR**	***P*-value^a^**
	**Mean or % (95% Cl)**	**Mean or % (95% Cl)**	**Mean or % (95% Cl)**	
N^b^	4426	3491	935	
Frequency (weighted) ^c^	18990825	14857122	4133703	
**Sociodemographic variables**				
Age (years)	60.7 ± 0.7	60.6 ± 0.8	61.0 ± 1.3	0.5790
Gender (male, %)	50.3 (48.0–52.6)	48.8 (46.2–51.5)	55.5 (50.8–60.3)	0.0174
**PIR (%)** ^**d**^				0.0322
<1.3 (low)	30.8 (28.2–33.4)	29.4 (26.8–32.1)	35.7 (30.3–41.1)	
1.3–4.9 (medium)	47.1 (44.8–49.5)	48.5 (46.1–50.9)	42.2 (36.6–47.7)	
5 (high)	13.0 (11.2–14.9)	13.4 (11.4–15.4)	11.7 (8.3–15.0)	
**Private insurance (%)** ^**e**^				0.0019
0	49.4 (46.9–51.9)	47.3 (44.5–50.1)	56.9 (52.1–61.7)	
1	47.5 (45.1–49.9)	49.4 (46.7–52.1)	40.7 (36.0–45.3)	
2	3.1 (2.2–4.0)	3.3 (2.3–4.3)	2.4 (0.8–4.0)	
**Clinical variables**				
SBP (mmHg)	131.1 ± 0.91	130.6 ± 1.0	133.2 ± 2.1	0.0212
Lymphocyte count (×10^9^/L)	2.3 ± 0.1	2.3 ± 0.1	2.1 ± 0.1	0.0004
Fasting glucose (mg/dL)	157.7 ± 3.0	153.9 ± 3.3	171.7 ± 6.8	<0.0001
HbA1c (%)	7.4 ± 0.1	7.3 ± 0.1	7.8 ± 0.2	<0.0001
ACR (mg/g)	160.9 ± 25.0	118.7 ± 19.3	312.3 ± 87.0	<0.0001
Family history (yes %)	69.7 (67.5–71.9)	68.2 (65.8–70.6)	75.0 (70.5–79.5)	0.0313
Duration of diabetes (years)	11.5 ± 0.46	10.3 ± 0.5	15.7 ± 1.1	<0.0001
Frequency of self–monitoring blood	1.9 ± 0.09	1.8 ± 0.1	2.2 ± 0.2	0.0062
**Treatment (%)**				<0.0001
Pills only	56.4 (53.9–58.8)	60.5 (57.9–63.2)	41.4 (36.3–46.5)	
Insulin only	13.4 (12.6–15.4)	10.0 (8.4 −11.7)	25.3 (21.2–29.5)	
Pills and insulin	14.0 (12.6–15.4)	11.6 (10.1–13.0)	22.6 (18.8–26.4)	
Neither	16.1 (14.3–17.9)	17.7 (15.7–19.7)	10.5 (6.8–14.3)	
**Comorbidities** ^**f**^				
HF (yes %)	10.4 (9.1–11.7)	8.6 (7.2–10.0)	17.1 (13.6–20.6)	0.0003
CHD (yes %)	12.0 (10.6–13.4)	10.7 (9.2–12.3)	16.6 (12.8–20.4)	0.0002
Heart attack ^e^ (yes %)	12.3 (10.7–13.8)	11.3 (9.7–12.9)	15.7 (12.0–19.4)	0.0029
Stroke (yes %)	10.3 (9.0– 11.7)	9.0 (7.7–10.3)	15.2 (11.5–19.0)	<0.0001
Liver condition (yes %)	9.1 (7.7–10.4)	7.8 (6.4–9.3)	13.5 (1.6–10.3)	0.0006
Renal failure (yes %)	9.5 (8.3–10.8)	6.8 (5.7–7.9)	19.4 (15.8–23.0)	<0.0001
**Depression (%)**				0.0007
None (0 to 4)	66.5 (64.3–68.7)	68.5 (66.0–70.9)	59.5 (55.1–64.0)	
Mild and moderate (5 to 14)	28.1 (25.9–30.2)	26.8 (24.4–29.1)	32.8 (28.6–37.0)	
Severe (≥15)	5.4 (4.5–6.3)	4.8 (3.8–5.8)	7.7 (5.2–10.1)	
**Behavioral variables**				
**Drink (%)**				0.0145
Being drinking	11.0 (9.6–12.4)	10.5 (9.1–11.9)	12.6 (9.3–15.9)	
Seldom	46.4 (43.3–49.5)	47.9 (44.6–51.1)	41.0 (35.8–46.3)	
Former	6.7 (5.5–8.0)	6.1 (4.9–7.3)	8.8 (5.7–11.9)	
Never	35.7 (33.0–38.5)	35.2 (32.2–38.2)	37.5 (32.8–42.2)	
Moderate recreational activities ^g^ (yes %)	34.2 (32.0–36.4)	35.4 (32.9–38.0)	29.8 (25.8–33.8)	0.0205
**Sleep hours (%)**				0.0054
<6h	16.2 (14.4–18.0)	15.2 (13.4–16.9)	20.0 (15.7–24.4)	
6 to 8h	65.0 (62.4–67.6)	67.0 (64.5–69.5)	57.8 (52.0–63.6)	
>8h	18.8 (15.9–21.6)	17.8 (15.2–20.4)	22.2 (16.3–28.1)	

Abbreviations: NHANES, National Health and Nutrition Examination Surveys; CI, confidence interval; DR, diabetic retinopathy; GED, General Educational Development; AA, Associate of Arts; PIR, Ratio of family income to poverty level; SBP, systolic blood pressure; HbA1c, glycosylated hemoglobin; ACR, urinary microalbumin/creatinine ratio; it was computed as albumin in milligrams per liter divided by creatinine in grams per liter; HF, Heart failure; CHD, coronary heart disease.

a This is a comparison between non–DR and DR adults having diabetes.

b The unweighted number of cases.

c All cases are weighted to be nationally representative.

d PIR was calculated by dividing the family income by the poverty guidelines specific to the survey year. The respondent only reported income as < $20,000 or ≥ $20,000, and the value was not computed.

e Number of private insurances covered by Medigap and single service plan.

f Doctors or health professionals diagnosed them.

g Sports, fitness, and recreational activities exclude the work and transport activities for at least 10 min continuously in a typical week.

It seems that diabetic relatives, duration of DM, frequency of self-monitoring blood glucose, pupils dilated exams, and insulin therapy indicated significant differences between the two groups. In addition, the DR group had significantly higher systolic blood pressure (133.3 ± 2.1 vs. 130.6 ± 1.0), blood levels of fasting glucose (171.7 ± 6.8 vs. 153.9 ± 3.3), glycosylated hemoglobin (7.8 ± 0.2 vs. 7.3 ± 0.1), the count of RBC (4.6 ± 0.1 vs. 4.6 ± 0.03), and a significantly higher urine albumin/creatinine ratio (312.3 ± 87.0 vs. 118.7 ± 19.3) (all *P* < 0.05). Otherwise, the count of lymphocytes (2.1 ± 0.1 vs. 2.3 ± 0.1) was significantly lower (all *P* < 0.05). Surprisingly, BMI, waist circumference, and diastolic blood pressure were not significantly different.

The DR group had more comorbidities. It had higher percentages of cardiovascular disease (HF, CHD, and MI), stroke, liver condition, and kidney failure than the non-DR group (all *P* < 0.05). In terms of psychological state, 40.5% of the participants with DR met the depression criteria based on their PHQ-9 questionnaire scores (32.8% vs. 26.8 in mild and moderate, 7.7% vs. 4.8 in severe group, *P* = 0.0007.

In the behavioral features, alcohol consumption and moderate recreational activities showed a significant difference between treatment groups (*P* = 0.0145 and 0.0205, respectively).

### Association of biological factors with DR

**Figure 2:**
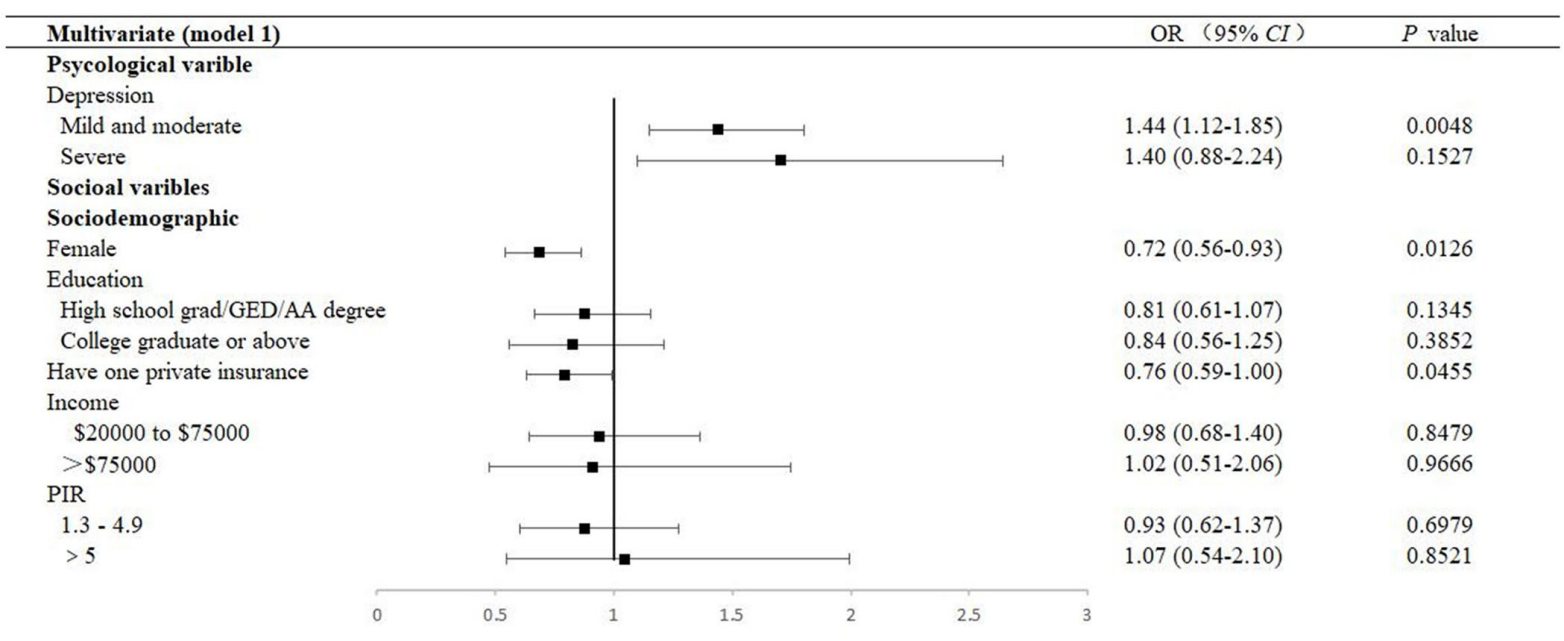
Multivariable forest plot of the association between factors and DR (Model 1). Model 1 was performed by adjusting sociodemographic variables. DR, diabetic retinopathy; OR, odds ratios; CI, confidence interval; GED, General Educational Development; AA, Associate of Arts; PIR, Ratio of family income to the poverty level, PIR was calculated by dividing family income by the poverty guidelines specific to the survey year, and the respondent only reported income as < $20,000 or ≥ $20,000, the value was not computed.

**Figure 3:**
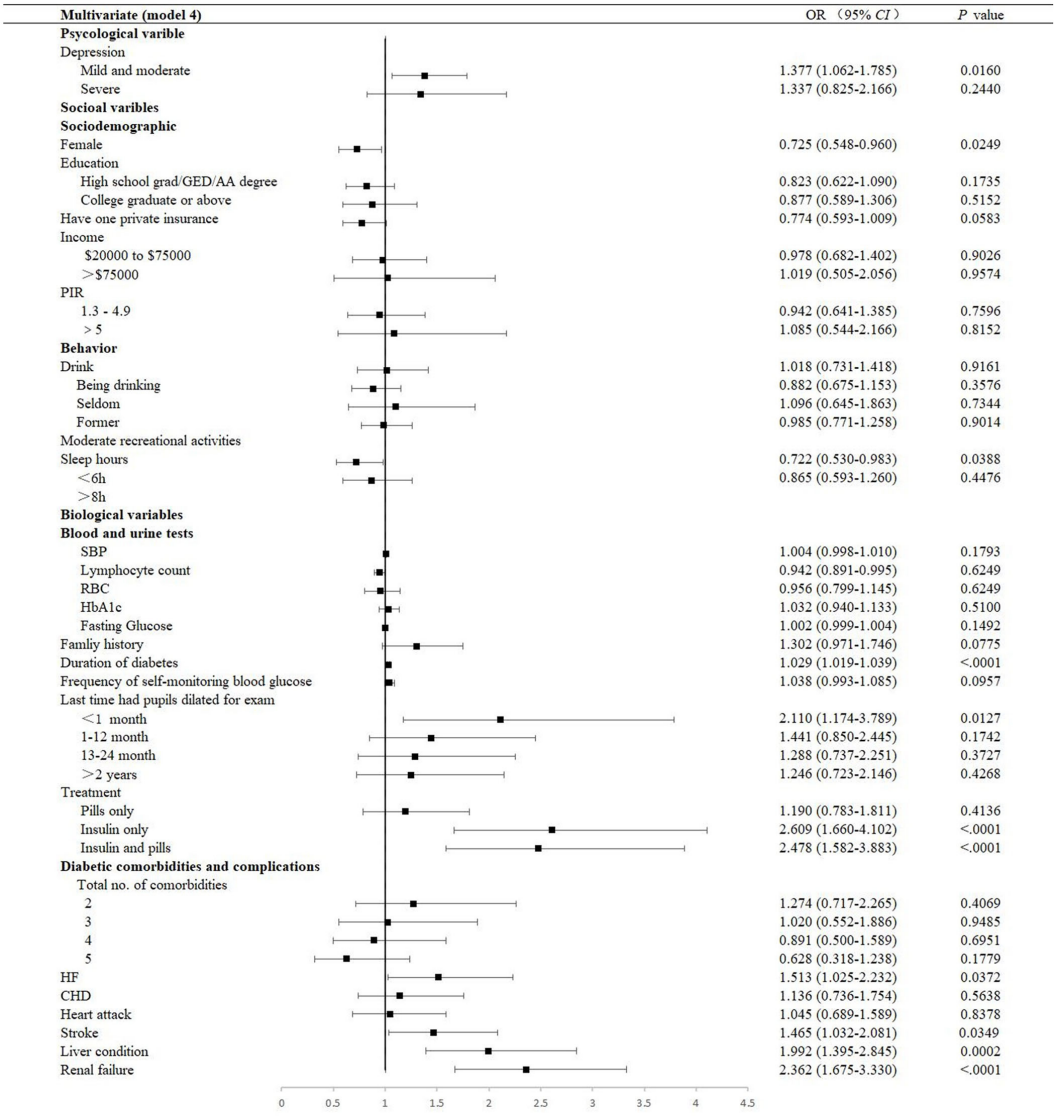
Multivariate forest plot of the association between factors and DR (Model 4). Model 4 was performed by adjusting all covariables, including sociodemographic variables, diabetic-related clinical variables, diabetic comorbidities, behavioral variables, and sociodemographic variables. DR, diabetic retinopathy; OR, odds ratios; CI, confidence interval; GED, General Educational Development; AA, Associate of Arts; PIR, Ratio of family income to the poverty level. PIR was calculated by dividing the family income by the poverty guidelines specific to the survey year; the respondent only reported income as < $20,000 or ≥ $20,000, and the value was not computed; SBP, systolic blood pressure; RBC, red blood cell; HbA1c, glycosylated hemoglobin; HF, heart failure; CHD, coronary heart disease.

The estimated ORs of univariate logistic regression and the descriptions carried out by the Chi-square tests were consistent. Factors with a *P*-value < 0.05 in the univariate analysis were selected for the multivariable analysis using the adjusted OR criterion to retain variables according to the univariate analysis (Figures 2, 3 and Supplementary Tables 5–8). It revealed that female sex (adjusted OR = 0.73, 95% CI: 0.55–0.94), having a low lymphocyte count (adjusted OR = 0.94, 95% CI: 0.89–0.99), longer duration of DM (adjusted OR = 1.03, 95% CI: 1.02–1.04), having pupils dilated exam within one month (adjusted OR = 2.11, 95% CI: 1.17–3.79), having received insulin treatment (insulin only, adjusted OR = 2.61, 95% CI: 1.66–4.10; pills and insulin, adjusted OR = 2.48, 95% CI: 1.58–3.88), and having comorbidities (HF, adjusted OR = 1.51, 95% CI: 1.03–2.23; stroke, adjusted OR = 1.47, 95% CI: 1.03–2.08; liver condition, OR = 1.99, 95% CI: 1.40–2.85; and renal failure, OR = 2.36, 95% CI: 1.68–3.33) were significantly associated with DR.

### Association of psychological factor with DR

The prevalence of mild and moderate depression of DR was 32.8% (95% CI: 28.6–37.0). Table 2 presents a stratified logistic regression analysis for depression and DR. In both the univariate and multivariate logistic regression models, we found that mild and moderate depression (adjusted OR = 1.38, 95% CI: 1.06–1.78, *P* = 0.0160) were independently associated with DR. However, in adjusted models, severe depression was no longer significant (Figures 2, 3 and Supplementary Figures 1, 2).

**Table 2:** Associations of depressive symptom severity and DR.

**OR (95% Cl)**	**Depressive symptom severity**				
	**None (0–4)**	**Mild and moderate (5–14)**	***P***	**Severe (≥15)**	***P***
Proportion (%)^a^	59.5	32.8		7.7	
Model 1^b^	Ref	1.440 (1.149 to 1.804)	0.0017	1.704 (1.100 to 2.640)	0.0173
Model 2^c^	Ref	1.504 (1.184 to 1.910)	0.0009	1.576 (1.006 to 2.470)	0.0473
Model 3^d^	Ref	1.437 (1.118 to 1.846)	0.0048	1.404 (0.881 to 2.238)	0.1527
Model 4^e^	Ref	1.377 (1.062 to 1.785)	0.0160	1.337 (0.825 to 2.166)	0.2377

a Prevalence of depressive symptom severity in DR.

b Model 1 adjusted for sociodemographic variables (gender, education, income, PIR, and private insurance).

c Model 2 adjusted for sociodemographic and diabetic-related clinical variables (systolic blood pressure, lymphocyte count, RBC, HbA1c%, fasting glucose, relatives having diabetes, duration of diabetes, frequency of self-monitoring of blood, the last time had pupils dilated for exams, and treatment).

d Model 3 adjusted for sociodemographic, diabetic-related clinical variables, and diabetic comorbidities and complications (the total number of comorbidities, HF, CHD, MI, stroke, liver condition, and renal failure).

e Model 4 adjusted for all covariables, including sociodemographic variables, diabetic-related clinical variables, comorbidities and complications, and behavioral variables (drinking, moderate recreational activities, and sleep hours).

### Association of behavioral factors with DR

As depicted, 9.3% and 29.8% participated in moderate and high-intensity recreational activities, respectively, among patients with DR. There was an association between behavioral factors, including alcohol consumption and moderate recreational activities (*P* = 0.0145 and 0.0205, respectively). However, in multivariate analysis, they were not identified as a significant indicator of DR (alcohol consumption, adjusted OR = 1.03, 95% CI: 0.74–1.43, and moderate recreational activities, adjusted OR = 0.98, 95% CI: 0.77–1.26, respectively). At the same time, the mean sleep duration was 7.08 ± 0.23 h a day, with an adjusted OR of 1.38 (95% CI: 1.01–1.88) in those with less than 6 h of sleep and 1.20 (95% CI: 0.88–1.64) in those with more than 8 h of sleep, compared to patients with 6–8 h of sleep (Figure 2, Supplementary Tables 3, 7).

## Discussion

The main objective of this study was to investigate the relationship between biological, social, and psychological risk factors and DR. In the analysis of biological factors, our findings confirmed that stroke was associated with DR, and the strength of the connection was not changed by controlling for confounders. On the contrary, retinal-related exams are expected to become a screening tool for brain lesions. We also found that mild and moderate depressions were independent risk factors for an increased prevalence of retinopathy in adult DM, but severe depression was not in the study of psychological factors. Furthermore, in the study of social behavioral factors, only a short sleep time may affect DR, while other behavioral factors, such as smoking and alcohol consumption, higher recreational activity, and less sedentary activity, had no effect on the progression of retinopathy in patients with diabetics.

It is necessary to use a public database to extensively investigate the risk factors of DR. Early evidence already showed a bidirectional relationship between depression and DM (7, 9). Nonetheless, these investigations have considered diabetes complications as a composite outcome and have given little attention to DR (31). Furthermore, most current studies have a small sample size, and qualitative research is limited to a specific state or ethnicity. To the best of our knowledge, our inquiry is the first to illustrate an overall picture of risk factors for DR using the NHANES database. Out study covers 50 states of the United States (30), has a professional survey organization, which is representative and reliable, and can also update, supplement, and verify the existing conclusions very well.

The indirect impact of depression on clinical risk factors should not be overlooked. A previous study (16) showed that psychological factors can directly affect the occurrence and development of DR by themselves and indirectly affect the process of DR through clinical characteristics. In terms of clinical factors, our findings support previous findings that a longer duration of DM (32–34) and insulin therapy were independently associated with DR (1). These correlations not only highlight that long illness or using insulin may heighten more severe diabetes and a higher risk of complications (35) but also suggest that negative emotion may lead to reduced willingness and worse adherence to treatment (11), poorer glycemic control (21, 36), and then exacerbating DR. Roy et al. (36) and Zou et al. (31) speculated that the hypothalamic-pituitary-adrenal (HPA) axis dysregulation is the cause of depression. It probably affects the pathogenesis of DR *via* hypercortisolemia and insulin resistance. Korczak et al. (37) and Wang et al. (38) added that circulating cytokine and insulin deficiency might explain blood glucose fluctuations, neurodevelopmental abnormalities, and neurocognitive deficits (7). In this study, after adjusting for confounding factors, the lymphocyte count in the DR group was still significantly higher than that in the non-DR group, which also provided evidence for the inflammatory mechanism. However, unlike previous studies (4, 32–34), we did not find a correlation between blood pressure, glycosylated hemoglobin, and DR in the multivariate regression analysis. It is most likely because over half of the respondents did not meet the standard for early morning fasting venous blood collection, which might lead to negative results.

The influence of psychological and behavioral factors on clinical indicators of DR should not be ignored when considering the biological, psychological, and social aspects of chronic diseases. Previous studies found that psychological variables are related to diabetic visual impairment, and the association between DR visual impairment and psychology deserves special clinical attention (10, 16). On this basis, we should fully apply the bio-psycho-social model of chronic disease to investigate the impact of psychological and behavioral factors on DR in the context of clinical factors.

In our study, 21.7% of DM had self-reported DR, which is lower than previous NHANES data (32.8%, 95% CI: 28.6–37.2) (1) but is not significantly different from the globally estimated prevalence of DR in the diabetic population (22.27%, 95% CI: 19.7–25.0) (39). This may be due to the fact that the previous database used retinal images of patients with DM over 40 years old as a reference, and the limitation of age and images improved the estimation of DR prevalence in the diabetic population. Our estimates from 2005 to 2018 indicated that there were 19 million of patients with DM in the United States, 4.1 million of whom were with DR, with the remainder having non-retinopathy, and this represents, on average, 0.1% of the entire population in this country. On the PHQ-9 questionnaire, 1.3 million (32.8%) and 0.3 million (7.7%) participants with DR between 5 and 14 scores and more than 15 scores have different severity of depressive symptoms.

Consistent with the conclusion from the critical meta-analysis of 11 cross-sectional and prospective cohort studies, it was found that depression is significantly associated with DR in patients with type 2 DM (31). Additionally, our results are in line with the results of Krystal Khoo et al., who found that DR is significantly associated with poor psychosocial outcomes after analyzing data from 42 studies (7). A 5-year prospective cohort study found that, for every significant 5-point increase in the severity level of depressive symptoms, the risk of incident DR would increase by approximately 15% (35). However, although our findings, like those of other studies, found that DR was independently linked with mild to moderate depression, we were the first to provide the conclusion that DR did not correlate with severe or major depression, which set us apart from the others. Why is there no correlation between major depression and DR? First, in the process of model adjustment, we noticed that the correlation between major depression status and DR disappeared when adjusting the related comorbidities and behavioral factors. We suspected that this might be due to the reason that DM associated with cardiac insufficiency, liver condition, kidney failure, etc., can also cause or aggravate the severe depressive state. Although the discrepancies between the univariate and multivariate logistic analyses of severe depression may account for collinearity or interaction between other risk factors and major depressive condition, there was a strong association between these comorbidities and DR, and the univariate association between depression and DR disappeared after adjustment for confounders. Second, a cross-sectional study from the Diabetes Management Project in the United Kingdom found that a history of depression or anxiety is the leading cause of DM accompanied by depression, and severe non-proliferative diabetic retinopathy (NPDR) or proliferative diabetic retinopathy (PDR) can only explain 19.1% of DM accompanied with depression symptoms (10). When other risk factors, such as the history of depression, were taken into account, major depression was uncorrelated with DR, and we hypothesize that the relationship between DR and severe depression may have been overestimated. Additionally, there may be a correlation between severe depressive state and DR. We have outstanding representative data for the American population, representing 1,027,913 DM with severe depression after weight. However, in terms of the proportion, severe depression accounted for only 5.4% of DM and 7.7% of DR in our study population. It is insufficient to analyze the potential correlation with a small proportion, covering up the possible correlation.

A preliminary study based on NHANES retinal images was presented at the American Stroke Association's International Stroke Congress in 2021. Retinal photographs may indicate an elevated risk of stroke, serving as an early warning signal for stroke prevention and treatment (40). Our study also discovered that DR is associated with stroke, and the association remains after adjusting for confounding factors. Similarly, a population-based cohort study found that retinal microvascular abnormalities provided a window into the brain for over a decade (41). There is a possibility for using retinal images as a screening tool to quickly screen out high-risk DR, compared to time-consuming and expensive magnetic resonance imaging (MRI). Although we examined the association between DR and stroke, more research is needed both on its causality and whether DR with depressive emotion will increase the risk of stroke so that they can have one more tool to screen for depression besides emotional scales.

Behavioral factors frequently coexist with depression to affect DM, and there are substantial differences in recreational activity and sleep between the DR and non-DR groups (13). According to the results, engaging in certain types of physical activities, such as exposure to sunlight and nature and social interactions, is associated with a decreased risk of depression and promotes resistance to stress (42). Studies revealed that moderate-to-vigorous physical activities are associated with DM *via* better glycemic control (43). Sedentary behavior and screen time levels, on the contrary, are associated with risk factors for chronic diseases, such as obesity, high fasting insulin levels, and metabolic syndrome during adolescence (44). A meta-analysis of 22 studies showed that moderate-intensity exercises were beneficial, while sedentary behavior increased DR risk significantly; nevertheless, the evidence was still insufficient (45), and findings are not convincingly consistent (46, 47). Interestingly, our study did not find positive results. We analyzed the association between the frequency of self-reported recreational activity and DR. First, as mentioned in reports (42), not all exercise alleviates depression. For DR with depression, concerns about hypoglycemia and retinal hemorrhage resulted in less high-intensity recreational activity in patients with DM and DR. Second, no difference in the HbA1c level between the DR and non-DR groups may indirectly reflect the same level of exercise between the two groups. Moreover, self-reporting produces a bias in representing actual physical activities, such as exercise types, duration, and intensity of recreational activity levels. All of these may affect the results.

Evidence on associations between sleep duration and DR is still lacking and inconsistent (17, 48). We observed the association between short sleep duration (≤6 h) and DR. It is possible that short and long sleep may have an influence on DR development through the disruption of circadian rhythm or abnormal glucose metabolism (12). Another NHANES study found that sleeping patterns might affect the psychological health (49). Similarly, intermittent hypoxia may increase the levels of vascular endothelial growth factors and other inflammatory cytokines that contribute to the progression of DR (50). However, our study found that sleeping trouble was not significantly associated with DR based on both self-reported and diagnosed data.

We cannot conclude that psychological or behavioral factors enhance the risk of DR or *vice versa*. In addition, interviews could not avoid certain recall biases. Furthermore, since DR diagnosis and severity are inaccurate, objective retinal imaging is required to determine whether depression is related to a specific subtype of DR. Thus, more profound observations and long-term cohort studies are necessary.

## Data availability statement

The raw data supporting the conclusions of this article will be made available by the authors, without undue reservation.

## Ethics statement

The studies involving human participants were reviewed and approved by Ethical approval for the use of the NHANES survey data from 2005 to 2010, 2011 to 2016 and 2017 to 2018 were obtained from the National Center for Health Statistics (NCHS) Research Ethics Review Board (ERB) through Protocol Number #2005-06, #2011-17 and #2018-01. All participants in this study were provided written informed consent. The information collected by the NCHS was kept with strict confidentiality bound to law. The patients/participants provided their written informed consent to participate in this study.

## Author contributions

X-JS was responsible for the statistical analysis and wrote the draft. G-HZ and C-MG assisted to collect the data. Z-YZ and Y-LN assisted to revise this draft. LW and G-RD designed this study. All authors contributed to the article and approved the submitted version.

## Funding

This research was supported by the National Natural Science Foundation of China (NSFC: 81970814, 81773553, 81670863), the Shaanxi Natural Science Basic Research Key Project (2021JZ-30), and the clinical AFFMU foundation support (2021JSTS28).

## Conflict of interest

The authors declare that the research was conducted in the absence of any commercial or financial relationships that could be construed as a potential conflict of interest.

## Publisher's note

All claims expressed in this article are solely those of the authors and do not necessarily represent those of their affiliated organizations, or those of the publisher, the editors and the reviewers. Any product that may be evaluated in this article, or claim that may be made by its manufacturer, is not guaranteed or endorsed by the publisher.
