# Supplementary material for: Associations between psycho-behavioral risk factors and diabetic retinopathy: NHANES (2005–2018)
Source: Front Public Health. 2022 Sep 15;10:966714. doi: 10.3389/fpubh.2022.966714 (PMC9521717; doi:10.3389/fpubh.2022.966714)
Supplement: Supplementary file 1 [file Data_Sheet_1.PDF]

eTable 1 Sociodemographic variables by diabetes in NHANES (2005-2018)

Variables	Total	Diabetes without DR	Diabetes with DR	*P*-value [a]
	Mean or % (*Cl* 95%)	Mean or % (*Cl* 95%)	Mean or % (*Cl* 95%)	
N [b]	4426	3491	935	
Frequency (weighted) [c]	18990825	14857122	4133703	
Age (years)	60.7 ± 0.7	60.6 ± 0.8	61.0 ± 1.3	0.5790
Gender (male, %)	50.3 (48.0-52.6)	48.8 (46.2-51.5)	55.5 (50.8-60.3)	0.0174
Race (%)				0.1115
Mexican American	15.4 (11.7-19.2)	15.3 (11.7-18.9)	16.1 (10.1-22.0)	
Other Hispanic	9.7 (7.6-11.8)	9.0 (7.0-11.0)	12.0 (7.8-16.1)	
Non-Hispanic white	41.7 (36.8-46.6)	43.1 (38.3-47.9)	36.7 (30.2-43.1)	
Non-Hispanic black	23.4 (19.7-27.0)	23.2 (19.5-27.0)	24.0 (19.0-28.9)	
Other/Mixed [d]	9.8 (7.9-11.8)	9.4 (7.5-11.3)	11.4 (7.8-14.9)	
Marital status (%)				0.8510
Married/ living with partners	60.8 (58.4-63.2)	60.9 (58.3-63.5)	60.3 (55.8.-64.7)	
Widowed/ divorced/ separate	30.4 (28.3-32.4)	30.1 (27.8-32.4)	31.3 (27.2-35.4)	
Never married	8.9 (7.5-10.2)	9.0 (7.5-10.4)	8.4 (5.6-11.3)	
Education (%)				0.0609
Less than High school grad	30.2 (27.7-32.6)	28.9 (26.3-31.5)	34.9 (29.5-40.3)	
High school grad/ GED/ AA	52.8 (50.3-55.4)	53.6 (50.8-56.4)	50.1 (44.4-55.9)	
College graduate or above	16.9 (15.0-18.7)	17.5 (15.4-19.5)	14.7 (10.7-18.7)	
Income (%)				0.0701
< $20000	23.9 (21.7-26.1)	22.9 (20.5-25.2)	27.5 (22.9-32.1)	
$20000 to $75000	47.1 (44.6-49.5)	47.8 (45.2-50.5)	44.3 (39.6-48.9)	
> $75000	19.3 (16.9-21.6)	19.9 (17.5-22.4)	16.9 (12.8-20.9)	
PIR (%)[e]				0.0322
< 1.3 (low)	30.8 (28.2-33.4)	29.4 (26.8-32.1)	35.7 (30.3-41.1)	
1.3 to 4.9 (medium)	47.1 (44.8-49.5)	48.5 (46.1-50.9)	42.2 (36.6-47.7)	
5 (high)	13.0 (11.2-14.9)	13.4 (11.4-15.4)	11.7 (8.3-15.0)	
Insurance (covered %)	87.8 (86.2-89.4)	87.9 (86.0-89.7)	87.4 (84.0-90.8)	0.8078
Private insurance (%) [f]				0.0019
0	49.4 (46.9-51.9)	47.3 (44.5-50.1)	56.9 (52.1-61.7)	
1	47.5 (45.1-49.9)	49.4 (46.7-52.1)	40.7 (36.0-45.3)	
2	3.1 (2.2-4.0)	3.3 (2.3-4.3)	2.4 (0.8-4.0)	

Abbreviations: NHANES, National Health and Nutrition Examination Surveys; *CI*, confidence interval; DR: diabetic retinopathy; GED, General Educational Development; AA, Associate of Arts; PIR, Ratio of family income to poverty level;

a. This is a comparison between non-DR and DR adults in diabetes.

b. Unweighted number of cases.

c. All cases are weighted to be nationally representative.

d. Multi-racial and Non-Hispanic Asian are included in the “Other/Mixed” group for non-Hispanic Asians fell into multi-racial categories before 2011.

e. PIR was calculated by dividing family income by the poverty guidelines specific to the survey year, and the respondent only reported income as < $20,000 or ≥ $20,000, the value was not computed.

g. Number of private insurances covered by Medi-Gap and single service plan.

eTable 2 Diabetic-related clinical variables by diabetes in NHANES (2005-2018)

Variables	Total	Diabetes without DR	Diabetes with DR	*P*-value [a]
	Mean or % (*Cl* 95%)	Mean or % (*Cl* 95%)	Mean or % (*Cl* 95%)	
N [b]	4426	3491	935	
Frequency (weighted) [c]	18990825	14857122	4133703	
BMI (kg/m^2) [d]	32.8 ± 0.37	33.0 ± 0.4	32.3 ± 0.7	0.0932
Waist circumference (cm)	110.5 ± 0.84	110.7 ± 0.9	109.5 ± 1.8	0.1756
SBP (mmHg)	131.1 ± 0.91	130.6 ± 1.0	133.2 ± 2.1	0.0212
DBP (mmHg)	69.2 ± 0.59	69.2 ± 0.6	69.6 ± 1.2	0.4641
Blood and urine tests				
Lymphocyte count ($\times 10^9$/L)	2.3 ± 0.1	2.3 ± 0.1	2.1 ± 0.1	0.0004
Red blood cell count ($\times 10^9$/L)	4.6 ± 0.0	4.6 ± 0.03	4.6 ± 0.1	0.0327
Fasting glucose (mg/dL)	157.7 ± 3.0	153.9 ± 3.3	171.7 ± 6.8	< 0.0001
HbA1c (%)	7.4 ± 0.1	7.3 ± 0.1	7.8 ± 0.2	< 0.0001
ACR (mg/g) [e]	160.9 ± 25.0	118.7 ± 19.3	312.3 ± 87.0	< 0.0001
Family history (yes %)	69.7 (67.5-71.9)	68.2 (65.8-70.6)	75.0 (70.5-79.5)	0.0313
Duration of diabetes (years)	11.5 ± 0.46	10.3 ± 0.5	15.7 ± 1.1	< 0.0001
Frequency of self-monitoring blood	1.9 ± 0.09	1.8 ± 0.1	2.2 ± 0.2	0.0062
Last time had pupils dilated for the exam (%)				0.0012
< 1 month	10.1 (8.8-11.3)	8.8 (7.3-10.2)	14.7 (11.5-17.9)	
1-12 months	53.7 (51.4-56.0)	53.4 (51.0-55.8)	55.0 (49.8-60.1)	
13-24 months	14.4 (12.8-16.1)	14.9 (13.0-16.8)	12.9 (9.4-16.4)	
> 2 years	14.4 (12.8-16.0)	14.9 (13.2-16.6)	12.5 (8.9-16.1)	
Never	6.7 (5.5 - 7.9)	7.3 (6.0 - 8.6)	4.3 (2.2- 6.4)	
Treatment (%)				< 0.0001
Pills only	56.4 (53.9-58.8)	60.5 (57.9-63.2)	41.4 (36.3-46.5)	
Insulin only	13.4 (12.6-15.4)	10.0 (8.4 -11.7)	25.3 (21.2-29.5)	
Pills and insulin	14.0 (12.6-15.4)	11.6 (10.1-13.0)	22.6 (18.8-26.4)	
Neither	16.1 (14.3-17.9)	17.7 (15.7-19.7)	10.5 (6.8-14.3)	

Abbreviations: NHANES, National Health and Nutrition Examination Surveys; *CI*, confidence interval; DR: diabetic retinopathy; BMI: body mass index; SBP: systolic blood pressure; DBP: diastolic blood pressure; HbA1c, glycosylated hemoglobin; ACR, urinary microalbumin/creatinine ratio;

a. This is a comparison between non-DR and DR adults in diabetes.

b. Unweighted number of cases.

c. All cases are weighted to be nationally representative.

d. BMI was computed as weight in kilograms divided by height in meters squared.

e. ACR was computed as albumin in milligrams per liter divided by creatinine in grams per liter.

eTable 3 Diabetic comorbidities and complications by diabetes in NHANES (2005-2018)

Variables	Total	Diabetes without DR	Diabetes with DR	*P*-value [a]
	Mean or % (*Cl* 95%)	Mean or % (*Cl* 95%)	Mean or % (*Cl* 95%)	
N [b]	4426	3491	935	
Frequency (weighted) [c]	18990825	14857122	4133703	
Comorbidities [d]				
Hypertension (yes %)	73.3 (71.3-75.3)	72.4 (70.3-74.6)	76.3 (72.2-80.4)	0.0954
High cholesterol (yes %)	62.4 (60.3-64.6)	61.4 (59.1-63.7)	66.1 (60.8-71.5)	0.1773
HF (yes %)	10.4 (9.1-11.7)	8.6 (7.2-10.0)	17.1 (13.6-20.6)	0.0003
CHD (yes %)	12.0 (10.6-13.4)	10.7 (9.2-12.3)	16.6 (12.8-20.4)	0.0002
Heart attack [e] (yes %)	12.3 (10.7-13.8)	11.3 (9.7-12.9)	15.7 (12.0-19.4)	0.0029
Angina (yes %)	8.0 (6.7- 9.3)	7.5 (6.0-8.9)	10.0 (7.0-13.1)	0.1660
Stroke (yes %)	10.3 (9.0- 11.7)	9.0 (7.7-10.3)	15.2 (11.5-19.0)	< 0.0001
Emphysema (yes %)	3.7 (2.9- 4.5)	3.8 (2.8-4.8)	3.3 (1.7-4.9)	0.7579
Chronic bronchitis (yes %)	10.5 (8.8- 12.1)	10.6 (8.8-12.4)	9.9 (6.7-13.0)	0.6998
Arthritis (yes %)	43.7 (40.7-46.6)	43.2 (40.1-46.3)	45.3 (40.4-50.1)	0.4884
Thyroid problem (yes %)	13.0 (11.5-14.4)	12.9 (0.8-11.4)	13.0 (1.6-9.9)	0.9949
Cancer or malignancy (yes %)	16.4 (14.4-18.3)	16.6 (14.6-18.5)	15.5 (11.5-19.5)	0.5170
Liver condition (yes %)	9.1 (7.7-10.4)	7.8 (6.4-9.3)	13.5 (1.6-10.3)	0.0006
Renal failure (yes %)	9.5 (8.3-10.8)	6.8 (5.7-7.9)	19.4 (15.8-23.0)	< 0.0001
Depression (%) [f]				0.0007
None (0 to 4)	66.5 (64.3-68.7)	68.5 (66.0-70.9)	59.5 (55.1-64.0)	
Mild and moderate (5 to 14)	28.1 (25.9-30.2)	26.8 (24.4-29.1)	32.8 (28.6-37.0)	
Severe (⩾15)	5.4 (4.5-6.3)	4.8 (3.8-5.8)	7.7 (5.2-10.1)	
Total no. of comorbidities (%)				0.0070
1	7.6 (6.3-9.0)	8.3 (6.8-9.7)	5.4 (2.8-8.0)	
2	24.6 (22.6-26.6)	25.0 (23.0-27.2)	23.1 (19.4-26.9)	
3	27.6 (25.8-29.4)	28.2 (26.2-30.2)	25.3 (21.0-29.6)	
4	17.3 (15.6-19.0)	17.3 (15.4-19.2)	17.2 (13.8-20.5)	
⩾5	22.9 (20.7-25.0)	21.2 (18.9-23.5)	29.0 (24.5-33.5)	

Abbreviations: NHANES, National Health and Nutrition Examination Surveys; *CI*, confidence interval; DR: diabetic retinopathy; HF: heart failure; CHD: coronary heart disease;

a. This is a comparison between non-DR and DR adults in diabetes.

b. Unweighted number of cases.

c. All cases are weighted to be nationally representative.

d. Doctors or health professionals diagnosed them.

e. Heart attack refers to myocardial infarction here.

f. Depressive symptoms measured using Patient Health Questionnaire-9.

eTable 4 Behavioral variables by diabetes in NHANES (2005-2018)

Variables	Total	Diabetes without DR	Diabetes with DR	*P*-value [a]
	Mean or % (*Cl* 95%)	Mean or % (*Cl* 95%)	Mean or % (*Cl* 95%)	
N [b]	4426	3491	935	
Frequency (weighted) [c]	18990825	14857122	4133703	
Smoke (%)				0.4277
Everyday	12.1 (10.6-13.6)	12.4 (10.7-14.1)	10.8 (8.0-13.7)	
Sometimes	3.2 (2.3-4.2)	2.9 (2.2-3.8)	4.2 (1.3-7.1)	
Never	84.7 (83.0-86.3)	84.6 (82.8-86.4)	84.9 (81.1-88.7)	
Drink (%)				0.0145
Being drinking	11.0 (9.6-12.4)	10.5 (9.1-11.9)	12.6 (9.3-15.9)	
Seldom	46.4 (43.3-49.5)	47.9 (44.6-51.1)	41.0 (35.8-46.3)	
Former	6.7 (5.5-8.0)	6.1 (4.9-7.3)	8.8 (5.7-11.9)	
Never	35.7 (33.0-38.5)	35.2 (32.2-38.2)	37.5 (32.8-42.2)	
Activities [d]				
Vigorous activities (yes %)	9.7 (8.2-11.3)	9.8 (8.3-11.4)	9.3 (5.9-12.6)	0.7455
Moderate activities (yes %)	34.2 (32.0-36.4)	35.4 (32.9-38.0)	29.8 (25.8-33.8)	0.0205
Days of activities per week	1.6 ± 0.12	1.6 ± 0.1	1.4 ± 0.2	0.0710
Minutes of activities per day [e]	34.1 ± 3.68	35.4 ± 4.0	29.6 ± 7.2	0.1320
Sedentary time [f]	380.5 ± 11.63	376.1 ± 12.6	396.0 ± 21.6	0.1264
Sleeping trouble (%)				0.4360
Doctor diagnosed	10.9 (9.0-12.7)	10.8 (8.9-12.6)	11.3 (8.1-14.4)	
Self-report	30.0 (27.4-32.5)	29.2 (26.4-32.0)	32.6 (27.7-37.4)	
Sleep hours (%)				0.0054
< 6h	16.2 (14.4-18.0)	15.2 (13.4-16.9)	20.0 (15.7-24.4)	
6 to 8h	65.0 (62.4-67.6)	67.0 (64.5-69.5)	57.8 (52.0-63.6)	
> 8h	18.8 (15.9-21.6)	17.8 (15.2-20.4)	22.2 (16.3-28.1)	

Abbreviations: NHANES, National Health and Nutrition Examination Surveys; *CI*, confidence interval; DR: diabetic retinopathy;

a. This is a comparison between non-DR and DR adults in diabetes.

b. Unweighted number of cases.

c. All cases are weighted to be nationally representative.

d. Sports, fitness, and recreational activities, exclude the work and transport activities for at least 10 minutes continuously in a typical week.

e. One minute of high-intensity activity is equal to two minutes of moderate -intensity activities, based on the database guidelines.

f. Sitting at school, at home, at a desk, sitting in a car or bus, reading books, playing cards, watching television, or using a computer. Exclude time spent sleeping on a typical day.

eTable 5 Regression of sociodemographic factors by diabetes in NHANES (2005-2018) [a]

Variables	Crude OR [b] (*Cl* 95%)	*P*-value	Adjusted OR [c] (*Cl* 95%)	*P*-value
Age	1.00 (0.99-1.01)	0.5824	----	----
Gender				
Male	Ref.		Ref.	
Female	0.77 (0.61-0.96)	0.0193	0.73 (0.55-0.94)	0.0249
Race				
Mexican American	Ref.		----	----
Other Hispanic	1.30 (0.78-2.04)	0.3475	----	----
Non-Hispanic white	0.81 (0.56-1.17)	0.2535	----	----
Non-Hispanic black	0.98 (0.67-1.43)	0.9244	----	----
Other/Mixed [d]	1.15 (0.72-1.82)	0.5586	----	----
Marital status				
Married or living with partners	Ref.		----	----
Widowed, divorced or separate	1.05 (0.85-1.30)	0.6530	----	----
Never married	0.95 (0.65-1.39)	0.7879	----	----
Education				
Less than High school grad	Ref.		Ref.	
High school grad/ GED/ AA degree	0.77 (0.59-1.02)	0.0702	0.82 (0.62-1.09)	0.1735
College graduate or above	0.70 (0.48-1.00)	0.0518	0.88 (0.59-1.31)	0.5152
Income				
< $20000	Ref.		Ref.	
$20000 to $75000	0.77 (0.59-1.00)	0.0489	0.98 (0.68-1.40)	0.9026
> $75000	0.70 (0.49-1.01)	0.0551	1.02 (0.51-2.06)	0.9574
PIR [e]				
< 1.3	Ref.		Ref.	
1.3-4.9	0.72 (0.55-0.94)	0.0162	0.94 (0.64-1.39)	0.7596
5	0.72 (0.50-1.03)	0.0696	1.09 (0.54-2.17)	0.8152
Insurance				
No	Ref.		----	----
yes	0.96 (0.67-1.36)	0.8063	----	----
Private insurance [f]				
0	Ref.		Ref.	
1	0.69 (0.55-0.85)	0.0006	0.77 (0.59-1.01)	0.0583
2	0.61 (0.30-1.23)	0.1664	0.82 (0.38-1.76)	0.6109

Abbreviations: NHANES, National Health and Nutrition Examination Surveys; CI, confidence interval; Ref., Reference; OR, odds ratio; GED, General Educational Development; AA, Associate of Arts; PIR, Ratio of family income to poverty level;

a. Data are weighted to representative US population

b. Unadjusted for univariate logistic regression of diabetes

c. Adjusted sociodemographic and clinical factors, comorbidities, and behavioural factors for multivariate logistic regression of diabetes

d. Multi-racial and Non-Hispanic Asian are included in the "Other/Mixed" group for non-Hispanic Asians fell into multi-racial categories before 2011.

e. PIR was calculated by dividing family income by the poverty guidelines specific to the survey year. The value was not computed f. The respondent only reported income as < $20,000 or ≥ $20,000.

f. Number of private insurances covered by Medi-Gap and single service plan.

eTable 6 Regression of clinical factors by diabetes in NHANES (2005-2018) [a]

Variables	Crude OR [b] (*Cl* 95%)	*P*-value	Adjusted OR [c] (*Cl* 95%)	*P*-value
BMI[d]	0.99 (0.98-1.00)	0.1033	----	----
Waist circumference	1.00 (0.99-1.00)	0.1812	----	----
SBP	1.01 (1.00-1.01)	0.0177	1.00 (1.00-1.01)	0.1793
DBP	1.00 (1.00-1.01)	0.4616	----	----
Blood and urine tests				
Lymphocyte count	0.91 (0.85-0.97)	0.0042	0.94 (0.89-0.99)	0.0342
Red blood cell count	0.83 (0.70-0.99)	0.0334	0.96 (0.80-1.15)	0.6249
Fasting glucose	1.00 (1.00-1.00)	<.0001	1.00 (1.00-1.00)	0.1492
HbA1c	1.15 (1.09-1.21)	<.0001	1.03 (0.94-1.13)	0.5100
Family history				
No	Ref.		Ref.	
Yes	0.78 (0.62-0.97)	0.0271	1.30 (0.97-1.75)	0.0775
Duration of diabetes	1.05 (1.03-1.06)	<.0001	1.03 (1.02-1.04)	<.0001
Frequency of self-monitoring blood	1.08 (1.01-1.14)	0.0150	1.04 (0.99-1.09)	0.0957
Last time had pupils dilated for the exam				
Never	Ref.		Ref.	
< 1 month	2.86 (1.60-5.12)	0.0004	2.11 (1.17-3.79)	0.0127
1-12 months	1.76 (1.03-3.00)	0.0380	1.44 (0.85-2.45)	0.1742
13-24 months	1.48 (0.90-2.44)	0.1221	1.29 (0.74-2.25)	0.3727
> 2 years	1.43 (0.82-2.52)	0.2094	1.25 (0.72-2.15)	0.4268
Treatment				
Neither	Ref.		Ref.	
Pills only	1.15 (0.74-1.78)	0.5390	1.19 (0.78-1.81)	0.4136
Insulin only	4.23 (2.65-6.77)	<.0001	2.61 (1.66-4.10)	<.0001
Pills and insulin	3.27 (2.10-5.09)	0.5654	2.48 (1.58-3.88)	<.0001

Abbreviations: NHANES, National Health and Nutrition Examination Surveys; CI, confidence interval; Ref., Reference; OR, odds ratio; BMI: body mass index; SBP: systolic blood pressure; DBP: diastolic blood pressure; HbA1c, glycosylated hemoglobin; ACR, urinary microalbumin/creatinine ratio;

a. Data are weighted to representative US population

b. Unadjusted for univariate logistic regression of diabetes

c. Adjusted sociodemographic and clinical factors, comorbidities, and behavioural factors for multivariate logistic regression of diabetes

d. BMI was computed as weight in kilograms divided by height in meters squared.

e. ACR was computed as albumin in milligrams per liter divided by creatinine in grams per liter.

eTable 7 Regression of comorbidities [d] by diabetes in NHANES (2005-2018) [a]

Variables	Crude OR [b] (*Cl* 95%)	*P*-value	Adjusted OR [c] (*Cl* 95%)	*P*-value
Hypertension				
No	Ref.		----	----
Yes	1.2 (0.96-1.56)	0.0964	----	----
High cholesterol				
No	Ref.		----	----
Yes	1.23 (0.96-1.59)	0.1064	----	----
HF				
No	Ref.		Ref.	
Yes	2.22 (1.64-3.00)	<.0001	1.51 (1.03-2.23)	0.0372
CHD				
No	Ref.		Ref.	
Yes	1.65 (1.19-2.27)	0.0026	1.14 (0.74-1.75)	0.5638
Heart attack [e]				
No	Ref.		Ref.	
Yes	1.46 (1.08-1.96)	0.0132	1.05 (0.69-1.59)	0.8378
Angina				
No	Ref.		----	----
Yes	1.39 (0.93-2.06)	0.1098	----	----
Stroke				
No	Ref.		Ref.	
Yes	1.82 (1.32-2.50)	0.0003	1.47 (1.03-2.08)	0.0329
Cancer or malignancy				
No	Ref.		----	----
Yes	0.92 (0.67-1.26)	0.6050	----	----
Liver condition				
No	Ref.		Ref.	
Yes	1.84 (1.31-2.58)	0.0005	1.99 (1.40-2.85)	0.0002
Renal failure				
No	Ref.		Ref.	
Yes	3.30 (2.49-4.38)	<.0001	2.36 (1.68-3.33)	<.0001
Depression [f]				
None	Ref.		Ref.	
Mild and moderate	1.41 (1.13-1.76)	0.0027	1.37 (1.06-1.79)	0.0160
Severe	1.85 (1.21-2.83)	0.0051	1.34 (0.83-2.17)	0.2440
Total no. of comorbidities				
1	Ref.		Ref.	
2	1.41 (0.82-2.44)	0.2119	1.27 (0.72-2.27)	0.4069
3	1.37 (0.76-2.46)	0.2887	1.02 (0.55-1.89)	0.9485
4	1.52 (0.88-2.63)	0.1364	0.89 (0.50-1.59)	0.6951
⩾5	2.10 (1.19-3.70)	0.0108	0.63 (0.32-1.24)	0.1779

Abbreviations: NHANES, National Health and Nutrition Examination Surveys; CI, confidence interval; Ref., Reference; OR, odds ratio; HF: heart failure; CHD: coronary heart disease;

a. Doctors or health professionals diagnosed them.

b. Data are weighted to representative US population

c. Unadjusted for univariate logistic regression of diabetes

d. Adjusted sociodemographic and clinical factors, comorbidities, and behavioural factors for multivariate logistic regression of diabetes

d. Heart attack refers to myocardial infarction here.

e. Depressive symptoms measured using Patient Health Questionnaire.

eTable 8 Regression of behavioural factors by diabetes in NHANES (2005-2018) [a]

Variables	Crude OR [b] (*Cl* 95%)	*P*-value	Adjusted OR [c] (*Cl* 95%)	*P*-value
Smoke				
Never	Ref.		----	----
Everyday	0.87 (0.64-1.21)	0.4100	----	----
Sometimes	1.42 (0.67-3.01)	0.3608	----	----
Drink				
Never	Ref.		Ref.	
Being drinking	1.12 (0.81-1.56)	0.4983	1.02 (0.73-1.42)	0.9161
Seldom	0.80 (0.64-1.02)	0.0687	0.88 (0.68-1.15)	0.3576
Former	1.34 (0.89-2.04)	0.1633	1.10 (0.65-1.86)	0.7344
Activities [c]				
Vigorous activities				
No	Ref.		----	----
Yes	0.94 (0.63-1.40)	0.7456	----	----
Moderate activities				
No	Ref.		Ref.	
Yes	0.77 (0.62-0.96)	0.0204	0.99 (0.77-1.26)	0.9014
Days of activities per week	0.96 (0.92-1.00)	0.0848	----	----
Minutes of activities per day [d]	1.00 (1.00-1.00)	0.1853	----	----
Sedentary time [e]	1.00 (1.00-1.00)	0.1324	----	----
Have Sleeping trouble				
No	Ref.		----	----
Doctor diagnosed	1.12 (0.82-1.53)	0.4642	----	----
Self-report	1.20 (0.93-1.54)	0.1694	----	----
Sleep hours				
6 to 8h	Ref.		Ref.	
< 6h	1.53 (1.14-2.05)	0.0044	1.38 (1.01-1.88)	0.0388
> 8h	1.45 (1.05-1.99)	0.0238	1.20 (0.88-1.64)	0.4476

Abbreviations: NHANES, National Health and Nutrition Examination Surveys; CI, confidence interval; Ref., Reference; OR, odds ratio;

a. Data are weighted to representative US population

b. Unadjusted for univariate logistic regression of diabetes

c. Adjusted sociodemographic and clinical factors, comorbidities, and behavioural factors for multivariate logistic regression of diabetes

d. Sports, fitness and recreational activities exclude the work and transport activities for at least 10 minutes continuously in a typical week.

e. One minute of high-intensity activity is equal to one minute of high-intensity activity, based on the database guidelines.

f. Sitting at school, at home, at a desk, travelling in a car or bus, reading, playing cards, watching television, or using a computer. Exclude time spent sleeping on a typical day.

eFigure 1 Multivariate forest plot of the association between factors and DR.

Multivariate (model 2)	OR （95% *CI*）	*P* value
**Psycological varible**		
Depression		
Mild and moderate	1.504 (1.184-1.910)	0.0009
Severe	1.576 (1.006-2.470)	0.0473
**Socioal varibles**		
**Sociodemographic**		
Female	0.666 (0.521-0.851)	0.0013
Education		
High school grad/GED/AA degree	0.806 (1.065-0.197)	0.1285
College graduate or above	0.827 (0.550-1.245)	0.3621
Have one private insurance	0.909 (0.430-1.924)	0.0234
Income		
$20000 to $75000	0.927 (0.648-1.325)	0.6745
＞$75000	0.960 (0.486-1.896)	0.9057
PIR		
1.3 - 4.9	0.888 (0.606-1.301)	0.5401
＞5	1.020 (0.511-2.038)	0.9549
**Biological variables**		
**Blood and urine tests**		
SBP	1.006 (1.000-1.012)	0.0456
Lymphocyte count	0.939 (0.886-0.995)	0.0344
RBC	0.876 (0.737-1.041)	0.1311
HbA1c %	1.012 (0.924-1.109)	0.7914
Fasting Glucose	1.002 (1.000-1.004)	0.1243
Famliy history	1.315 (0.992-1.744)	0.0568
Duration of diabetes	1.028 (1.018-1.039)	<.0001
Frequency of self-monitoring blood glucose	1.034 (0.998-1.083)	0.1472
Last time had pupils dilated for exam		
＜1 month	2.031 (1.119-3.686)	0.0201
1-12 month	1.371 (0.819-2.297)	0.2290
13-24 month	1.253 (0.755-2.080)	0.3814
＞2 years	1.268 (0.727-2.211)	0.4016
Treatment		
Pills only	1.104 (0.731-1.667)	0.6363
Insulin only	2.890 (1.829-4.566)	<.0001
Insulin and pills	2.402 (1.520-3.795)	0.0002

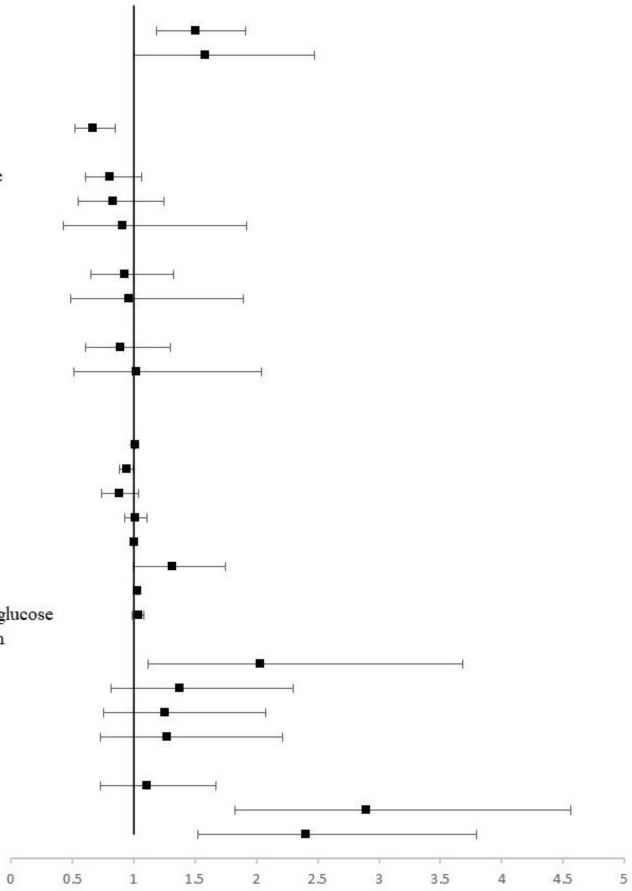

Model 2 was performed by adjusting sociodemographic and diabetic-related clinical variables including gender, education, income, PIR, private insurance, SBP, lymphocyte count, RBC, HbA1c %, fasting glucose, relatives have diabetes, duration of diabetes, frequency of self-monitoring blood, last time had pupils dilated for exams, and treatment. DR: diabetic retinopathy; OR, odds ratios; *CI*, confidence interval; GED, General Educational Development; AA, Associate of Arts; PIR, Ratio of family income to the poverty level, PIR was calculated by dividing family income by the poverty guidelines specific to the survey year, and the respondent only reported income as < $20,000 or ≥ $20,000, the value was not computed; SBP: systolic blood pressure; RBC: red blood cell; HbA1c, glycosylated hemoglobin.

eFigure 2 Multivariate forest plot of the association between factors and DR.

Multivariate (model 3)	OR （95% *CI*）	*P* value
**Psycological varible**		
Depression		
Mild and moderate	1.437(1.118-1.846)	0.0048
Severe	1.404(0.881-2.238)	0.1527
**Socioal varibles**		
**Sociodemographic**		
Female	0.721 (0.558-0.932)	0.0126
Education		
High school grad/GED/AA degree	0.809 (0.612-1.068)	0.1345
College graduate or above	0.838 (0.561-1.251)	0.3852
Have one private insurance	0.764 (0.587-0.995)	0.0455
Income		
$20000 to $75000	0.965 (0.671-1.389)	0.8479
＞$75000	0.985 (0.496-1.959)	0.9666
PIR		
1.3 - 4.9	0.925 (0.624-1.371)	0.6976
> 5	1.066 (0.541-2.103)	0.8521
**Biological variables**		
**Blood and urine tests**		
SBP	1.004 (0.998-1.010)	0.1884
Lymphocyte count	0.943 (0.891-0.997)	0.0389
RBC	0.956 (0.798-1.145)	0.6258
HbA1c %	1.032 (0.940-1.133)	0.5042
Fasting Glucose	1.002 (1.000-1.004)	0.1326
Famliy history	1.296 (0.970-1.733)	0.0791
Duration of diabetes	1.029 (1.019-1.040)	<.0001
Frequency of self-monitoring blood glucose	1.037 (0.992-1.083)	0.1074
Last time had pupils dilated for exam		
＜1 month	2.116 (1.191-3.759)	0.0108
1-12 month	1.453 (0.866-2.438)	0.1563
13-24 month	1.306 (0.750-2.272)	0.3436
＞2 years	1.261 (0.735-2.164)	0.3972
Treatment		
Pills only	1.181 (0.777-1.794)	0.4353
Insulin only	2.593 (1.655-4.063)	<.0001
Insulin and pills	2.465 (1.578-3.850)	<.0001
**Diabetic comorbidities and complications**		
Total no. of comorbidities		
2	1.279 (0.710-2.306)	0.4108
3	1.040 (0.557-1.940)	0.9024
4	0.910 (0.505-1.639)	0.7516
5	0.651 (0.328-1.289)	0.2169
HF	1.510 (1.026-2.224)	0.0369
CHD	1.126 (0.728-1.741)	0.5913
Heart attack	1.057 (0.697-1.602)	0.7928
Stroke	1.482 (1.041-2.108)	0.0290
Liver condition	1.997 (1.384-2.882)	0.0003
Renal failure	2.386 (1.703-3.344)	<.0001

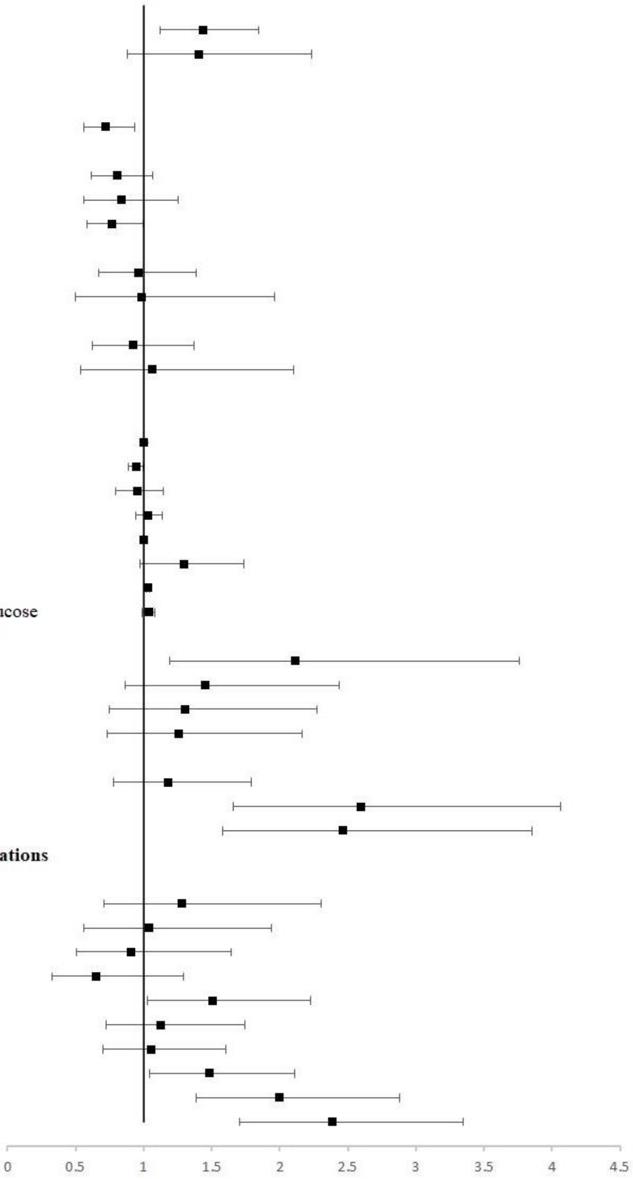

Model 3 was performed by adjusting sociodemographic and biological(diabetic-related clinical variables and diabetic comorbidities and complications) factors, including gender, education, income, PIR, private insurance, SBP, lymphocyte count, RBC, HbA1c %, fasting glucose, relatives have diabetes, duration of diabetes, frequency of self-monitoring blood, last time had pupils dilated for exams, treatment, the total number of comorbidities, HF, CHD, heart attack, stroke, liver condition, and renal failure. DR: diabetic retinopathy; OR, odds ratios; *CI*, confidence interval; GED, General Educational Development; AA, Associate of Arts; PIR, Ratio of family income to the poverty level, PIR was calculated by dividing family income by the poverty guidelines specific to the survey year, and the respondent only reported income as < $20,000 or ≥ $20,000, the value was not computed; SBP: systolic blood pressure; RBC: red blood cell; HbA1c, glycosylated hemoglobin; HF: heart failure; CHD: coronary heart disease.
